# Using Genetic Distance to Infer the Accuracy of Genomic Prediction

**DOI:** 10.1371/journal.pgen.1006288

**Published:** 2016-09-02

**Authors:** Marco Scutari, Ian Mackay, David Balding

**Affiliations:** 1 Department of Statistics, University of Oxford, Oxford, United Kingdom; 2 National Institute of Agricultural Botany (NIAB), Cambridge, United Kingdom; 3 Centre for Systems Genomics, School of BioSciences and of Mathematics and Statistics, University of Melbourne, Melbourne, Australia; 4 Genetics Institute, University College London (UCL), London, United Kingdom; The Roslin Institute, The University of Edinburgh, UNITED KINGDOM

## Abstract

The prediction of phenotypic traits using high-density genomic data has many applications such as the selection of plants and animals of commercial interest; and it is expected to play an increasing role in medical diagnostics. Statistical models used for this task are usually tested using cross-validation, which implicitly assumes that new individuals (whose phenotypes we would like to predict) originate from the same population the genomic prediction model is trained on. In this paper we propose an approach based on clustering and resampling to investigate the effect of increasing genetic distance between training and target populations when predicting quantitative traits. This is important for plant and animal genetics, where genomic selection programs rely on the precision of predictions in future rounds of breeding. Therefore, estimating how quickly predictive accuracy decays is important in deciding which training population to use and how often the model has to be recalibrated. We find that the correlation between true and predicted values decays approximately linearly with respect to either *F*_ST_ or mean kinship between the training and the target populations. We illustrate this relationship using simulations and a collection of data sets from mice, wheat and human genetics.

## Author Summary

The availability of increasing amounts of genomic data is making the use of statistical models to predict traits of interest a mainstay of many applications in life sciences. Applications range from medical diagnostics for common and rare diseases to breeding characteristics such as disease resistance in plants and animals of commercial interest. We explored an implicit assumption of how such prediction models are often assessed: that the individuals whose traits we would like to predict originate from the same population as those that are used to train the models. This is commonly not the case, especially in the case of plants and animals that are parts of selection programs. To study this problem we proposed a model-agnostic approach to infer the accuracy of prediction models as a function of two common measures of genetic distance. Using data from plant, animal and human genetics, we find that accuracy decays approximately linearly in either of those measures. Quantifying this decay has fundamental applications in all branches of genetics, as it measures how studies generalise to different populations.

## Introduction

Predicting unobserved phenotypes using high-density SNP or sequence data is the foundation of many applications in medical diagnostics [1–3], plant [4, 5] and animal [6] breeding. The accuracy of genomic predictions will depend on a number of factors: relatedness among genotyped individuals [7, 8]; the density of the markers [7, 9, 10]; and the genetic architecture of the trait, in particular the allele frequencies of causal variants [11, 12] and the distribution of their effect sizes [7].

Most of these issues have been explored in the literature, and have been tackled in various ways either from a methodological perspective or by producing larger data sets and more accurate phenotyping. However, the extent to which predictive models generalise from the populations used to train them to distantly related target populations appears not to have been widely investigated (two exceptions are [7, 13]). The accuracy of prediction models is often evaluated in a general setting using cross-validation with random splits, which implicitly assumes that test individuals are drawn from the same population as the training sample; in that case accuracy to predict phenotypes is only bounded by heritability, although unaccounted “missing heritability” is common [14, 15]. However, this assumption is violated in many practical applications, such as genomic selection, that require predictions of individuals that are genetically distinct from the training sample: for instance, causal variants may differ in both frequency and effect size between different ancestry groups (in humans, e.g. [16] for lactose persistence), subspecies (in plants and animals, e.g. [17] for rice) or even families [18]. In such cases cross-validation with random splits may overestimate predictive accuracy due to the mismatch between model validation and the prediction problem of interest [19, 20] even when population structure is taken into account [21]. The more distantly the target population is related to the training population, the lower the average predictive accuracy of a genomic model; this has been demonstrated on both simulated and real dairy cattle data [20, 22, 23].

In this paper we will investigate the relationship between genetic distance and predictive accuracy in the prediction of quantitative traits. We will simulate training and target samples with varying genetic distances by splitting the training population into a sequence of pairs of subsets with increasing genetic differentiation. We will measure predictive accuracy with Pearson’s correlation, which we will estimate by performing genomic prediction from one subset to the other in each pair. Among various measures of relatedness available in the literature, we will consider mean kinship and *F*_ST_, although we will only focus on the latter. We will then study the mean Pearson’s correlation as a function of genetic distance, which we will refer to as the “decay curve” of the former over the latter.

This approach is valuable in addressing several key questions in the implementation of genomic selection programs, such as: How often (e.g., in terms of future generations) will the genomic prediction model have to be re-estimated to maintain a minimum required accuracy in the predictions of the phenotypes? How should we structure our training population to maximise that accuracy? Which new, distantly related individuals would be beneficial to introduce in a selection program for the purpose of maintaining a sufficient level of genetic variability?

## Materials and Methods

### Genomic Prediction Models

A baseline model for genomic prediction of quantitative traits is the genomic BLUP (GBLUP; [24, 25]), which is usually written as

$$y=\mu +\text{Zg}+\varepsilon \;\text{with}\;g∼N\left(0,K\sigma_{g}^{2}\right)\;\text{and}\;\varepsilon ∼N\left(0,\sigma_{\varepsilon }^{2}\right),$$ (1)

where **g** is a vector of genetic random effects, **Z** is a design matrix that can be used to indicate the same genotype exposed to different environments, **K** is a kinship matrix and *ε* is the error term. Many of its properties are available in closed form thanks to its simple definition and normality assumptions, including closed form expressions of and upper bounds on predictive accuracy that take into account possible model misspecification [15]. Other common choices are additive linear regression models of the form

y=μ+Xβ+ε (2)

where **y** is the trait of interest; **X** are the markers (such as SNP allele counts coded as 0, 1 and 2 with 1 the heterozygote); ***β*** are the marker effects; and *ε* are independent, normally-distributed errors with variance $\sigma_{\varepsilon }^{2}$. Depending on the choice of the prior distribution for ***β***, we can obtain different models from the literature such as BayesA and BayesB [25], ridge regression [26], the LASSO [27] or the elastic net [28]. The model in Eq (1) is equivalent to that in Eq (2) if the kinship matrix **K** is computed from the markers **X** and has the form **X** **X**^*T*^ and ***β*** ∼ *N*(0, VAR(***β***)) [29, 30]. In the remainder of the paper we will focus on the elastic net, which we have found to outperform other predictive models on real-world data [31]. This has been recently confirmed in [32].

Predictive accuracy is often measured by the Pearson correlation ($\hat{\rho }$) between the predicted and observed phenotypes. When we use the fitted values from the training population as the predicted phenotypes, and assuming that the model is correctly specified, ${\hat{\rho }}^{2}$ coincides with the proportion of genetic variance of the trait explained by the model and therefore ${\hat{\rho }}^{2}⩽h^{2}$, the heritability of the trait. (An incorrect model may lead to overfitting, and in that case ${\hat{\rho }}^{2}⩾h^{2}$.) When using cross-validation with random splits, ${\hat{\rho }}_{\text{CV}}⩽\hat{\rho }$ and typically the difference will be noticeable (${\hat{\rho }}_{\text{CV}}\ll \hat{\rho }$). However, ${\hat{\rho }}_{CV}$ may still overestimate the actual predictive accuracy ${\hat{\rho }}_{D}$ in practical applications where target individuals for prediction are more different from the training population than the test samples generated using cross-validation [14]. This problem may be addressed by the use of alternative model validation schemes that mirror more closely the prediction task of interest; for instance, by simulating progeny of the training population to assess predictive accuracy for a genomic selection program. This approach is known as forward prediction and is common in animal breeding [19, 33].

Another possible choice is the prediction error variance (PEV). It is commonly used in conjunction with GBLUP because, for that model, it can be estimated (for small samples) or approximated (for large samples) in closed form from Henderson’s mixed model equations [34]. In the general case no closed form estimate is available, but PEV can still be derived from Pearson’s correlation [35] for any kind of model as both carry the same information:

$$\text{PEV}=\left(1-{\hat{\rho }}^{2}\right)*\mathrm{VAR}\left(y\right).$$ (3)

For consistency with our previous work [31] and with [4], whose results we partially replicate below, we will only consider predictive correlation in the following.

### Kinship Coefficients and *F*_ST_

A common measure of kinship from marker data is average allelic correlation [24, 36], which is defined as **K** = [*k*_*ij*_] with

$$k_{ij}=\frac{1}{m}\sum_{k=1}^{m}{\tilde{X}}_{ik}{\tilde{X}}_{jk}$$ (4)

where ${\tilde{X}}_{ik}$ and ${\tilde{X}}_{jk}$ are the standardised allele counts for the *i*th and *j*th individuals and the *k*th marker. An important property of allelic correlation is that it is inversely proportional to the Euclidean distance between the marker profiles *X*_*i*_, *X*_*j*_ of the corresponding individuals: if the markers are standardised

$$\sqrt{2n-2k_{ij}}=\sqrt{2n-2\sum_{k=1}^{m}{\tilde{X}}_{ik}{\tilde{X}}_{jk}}=\sqrt{\sum_{k=1}^{m}{\tilde{X}}_{ik}^{2}+{\tilde{X}}_{jk}^{2}-2{\tilde{X}}_{ik}{\tilde{X}}_{jk}}=\sqrt{\sum_{k=1}^{m}{\left({\tilde{X}}_{ik}-{\tilde{X}}_{jk}\right)}^{2}}.$$ (5)

This result has been used in conjunction with clustering methods such as *k*-means or partitioning around medoids (PAM; [37]) to produce subsets of minimally related individuals from a given sample by maximising the Euclidean distance [14, 19, 38].

At the population level, the divergence between two populations due to drift, environmental adaptation, or artificial selection is commonly measured with *F*_ST_. Several estimators are available in the literature, and reviewed in [39]. In this paper we will adopt the estimator from [40], which is obtained by maximising the Beta-Binomial likelihood of the allele frequencies as a function of *F*_ST_. ${\hat{F}}_{\text{ST}}$ then describes how far the target population has diverged from the training population, which translates to “how far” a genomic prediction model will be required to predict. In terms of kinship, we know from the literature that the mean kinship coefficient $\bar{k}$ between two individuals in different populations is inversely related to ${\hat{F}}_{\text{ST}}$ [41]: kinship can be interpreted as the probability that two alleles are identical by descent, which is inversely related to *F*_ST_ which is a mean inbreeding coefficient. Intuitively, the fact that individuals in the two populations are closely related implies that the latter have not diverged much from the former: if $\bar{k}$ is large, the marker profiles (and therefore the corresponding allele frequencies) will on average be similar. As a result, any clustering method that uses the Euclidean distance to partition a population into subsets will maximise their *F*_ST_ by minimising $\bar{k}$. The simulations and data analyses below confirm experimentally that $\bar{k}$ and ${\hat{F}}_{\text{ST}}$ are highly correlated, which makes them equivalent in building the decay curves; thus we will report results only for ${\hat{F}}_{\text{ST}}$ (see Section C in S1 Text).

### Real-World Data Sets

We evaluate our approach to construct decay curves for predictive accuracy using two publicly-available real-world data sets with continuous phenotypic traits, and a third, human, genotype data set.

#### WHEAT

We consider 376 wheat varieties from the TriticeaeGenome project, described in [4]. Varieties collected from those registered in France (210 varieties), Germany (90 varieties) and the UK (75 varieties) between 1946 and 2007 were genotyped using a combination of 2712 predominantly DArT markers. Several traits were recorded; in this paper we will focus on grain yield, height, flowering time, and grain protein content. Genotype-environment interactions were accounted for by an incomplete block design over trial fields in different countries, to prevent genomic prediction being biased by the country of registration of each variety. As in [4], we also group varieties in three groups based on their year of registration: pre-1990 (103 varieties), 1990 to 1999 (120 varieties), and post-1999 (153 varieties).

#### MICE

The heterogeneous mice population from [42] consists of 1940 individuals genotyped with 12545 SNPs; among the recorded traits, we consider growth rate and weight. The data include a number of inbred families, the largest being F005 (287 mice), F008 (293 mice), F010 (332 mice) and F016 (309 mice).

#### HUMAN

The marker profiles from the Human Genetic Diversity Panel [43] include 1043 individuals from different ancestry groups: 151 from Africa, 108 from America, 435 from Asia, 167 from Europe, 146 from the Middle East and 36 from Oceania. Each has been genotyped with 650,000 SNPs; for computational reasons we only use those in chromosomes 1 and 2, for a total of 90,487 SNPs.

All data sets have been pre-processed by removing markers with minor allele frequencies < 1% and those with > 20% missing data. The missing data in the remaining markers have been imputed using the **impute** R package [44]. Finally, we removed one marker from each pair whose allele counts have correlation > 0.95 to increase the numerical stability of the genomic prediction models.

### Decay Curves for Predictive Accuracy

We estimate a decay curve of ${\hat{\rho }}_{D}$ as a function of *F*_ST_ as follows:

1. Produce a pair of minimally related subsets (i.e., with maximum *F*_ST_) from our training population using *k*-means clustering, *k* = 2 in R [45]. PAM was also considered as an alternative clustering method, but produced subsets identical to those from *k*-means for all the data sets studied in this paper. The largest of these two subsets will be used to train the genomic prediction model, and will be considered the ancestral population for the purposes of computing *F*_ST_; the smallest will be the target used for prediction. In the following we will call them the *training subsample* and the *target subsample*, respectively.
2. Compute ${\hat{F}}_{\text{ST}}^{\left(0\right)}$ and ${\hat{\rho }}_{D}^{\left(0\right)}$ for the pair of subsets with a genomic prediction model. We compute ${\hat{F}}_{\text{ST}}^{\left(0\right)}$ using the Beta-Binomial estimator from [40]; and we compute ${\hat{\rho }}_{D}^{\left(0\right)}$ with the elastic net implementation in the **glmnet** R package [46]. Other models can be used: the proposed approach is model-agnostic as it only requires the chosen model to be able to produce estimates of its predictive correlation. The optimal values for the penalty parameters of the elastic net are chosen to maximise ${\hat{\rho }}_{\text{CV}}$ on the training subset using 5 runs of 10-fold cross-validation as in [47]. $\left({\hat{F}}_{\text{ST}}^{\left(0\right)},{\hat{\rho }}_{D}^{\left(0\right)}\right)$ will act as the far end of the decay curve (in terms of genetic distance).
3. For increasing numbers *m* of individuals:
  - create a new pair of subsamples by swapping *m* individuals at random between the training and the test subsamples from step 1;
  - fit a genomic prediction model on the new training subsample and use it to predict the new target subsample, thus obtaining $\left({\hat{F}}_{\text{ST}}^{\left(m\right)},{\hat{\rho }}_{D}^{\left(m\right)}\right)$ using the same algorithms as in step 2.
4. Estimate the decay curve from the sets of $\left({\hat{F}}_{\text{ST}}^{\left(m\right)},{\hat{\rho }}_{D}^{\left(m\right)}\right)$ points using local regression (LOESS; [48]), which can be used to produce both the mean and its 95% confidence interval at any point in the range of observed ${\hat{F}}_{\text{ST}}$. We denote with ${\hat{\rho }}_{D}$ the resulting estimate of predictive correlation for any given ${\hat{F}}_{\text{ST}}$.

The pair of subsets produced by *k*-means corresponds to *m* = 0, hence the notation $\left({\hat{F}}_{\text{ST}}^{\left(0\right)},{\hat{\rho }}_{D}^{\left(0\right)}\right)$, and we increase *m* by steps of 2 to 20 until the ${\hat{F}}_{\text{ST}}$ between the subsamples is at most 0.005. We choose the stepping for each data set to be sufficiently small to cover the interval $\left[0,{\hat{F}}_{\text{ST}}^{\left(0\right)}\right]$ as uniformly as possible. The larger *m* is, the smaller we can expect ${\hat{F}}_{\text{ST}}^{\left(m\right)}$ to be. We repeat step 3(a) and 3(b) 40 times for each *m* to achieve the precision needed for an acceptably smooth curve.

As an alternative approach, we also consider estimating the decay rate of ${\hat{\rho }}_{D}$ by linear regression of the ${\hat{\rho }}_{D}^{\left(m\right)}$ against the ${\hat{F}}_{\text{ST}}^{\left(m\right)}$; we will denote the resulting predictive accuracy estimates with ${\hat{\rho }}_{L}$. For any set value of ${\hat{F}}_{\text{ST}}$, we compare the ${\hat{\rho }}_{L}$ at that ${\hat{F}}_{\text{ST}}$ with the corresponding value ${\hat{\rho }}_{D}$ from the decay curve estimated by averaging all the ${\hat{\rho }}_{D}^{\left(m\right)}$ for which $|{\hat{F}}_{\text{ST}}^{\left(m\right)}-{\hat{F}}_{\text{ST}}|⩽0.01$. Assuming that the decay curve is in fact a straight line reduces the number of subsamples that we need to generate, enforces smoothness and makes it possible to compute ${\hat{\rho }}_{L}$ for values of *F*_ST_ larger than ${\hat{F}}_{\text{ST}}^{\left(0\right)}$. On the other hand, the estimated ${\hat{\rho }}_{L}$ will be increasingly unreliable as ${\hat{\rho }}_{L}\to 0$, because the regression line will provide negative ${\hat{\rho }}_{L}$ instead of converging asymptotically to zero. We also regress the ${\left({\hat{\rho }}_{D}^{\left(m\right)}\right)}^{2}$ against the ${\left({\hat{F}}_{\text{ST}}^{\left(m\right)}\right)}^{2}$ to investigate whether they have a stronger linear relationship than the ${\hat{\rho }}_{D}^{\left(m\right)}$ with the ${\hat{F}}_{\text{ST}}^{\left(m\right)}$, as suggested in [22] using simulated genotypes and phenotypes mimicking a dairy cattle population.

The size of the training (*n*_TR_) and target (*n*_TA_) subsamples is determined by *k*-means. For the data used in this paper, *k*-means splits the training populations in two subsamples of comparable size; but we may require a smaller *n*_TA_ ≪ *n*_TR_ to estimate ${\hat{\rho }}_{D}^{\left(0\right)}$ and the ${\hat{\rho }}_{D}^{\left(m\right)}$ while at the same time a larger *n*_TR_ is needed to fit the genomic prediction model. In that case, we increase *n*_TR_ by moving individuals from the target subsample while keeping the ${\hat{F}}_{\text{ST}}^{\left(0\right)}$ between the two as large as possible. The impact on the estimated ${\hat{F}}_{\text{ST}}$ is likely to be small, because its precision depends more on the number of markers than on *n*_TR_ and *n*_TA_ [40]. The estimated ${\hat{\rho }}_{D}^{0}$ and ${\hat{\rho }}_{D}^{\left(m\right)}$ might be inflated because we are altering the subsets, even when ${\hat{F}}_{\text{ST}}$ does not change appreciably. Its variance, which can be approximated as in [49], decreases linearly in *n*_TA_ except that can be compensated by generating more pairs of subsamples for each value of *m*.

### Simulation Studies

We study the behaviour of the decay curves via two simulation studies.

#### Genomic selection

We simulate a genomic selection program using the wheat varieties registered in the last 5 years of the WHEAT data as founders. The simulation is a forward simulation implemented as follows for 10, 50, 200 and 1000 causal variants, and decay curves are produced for each.

1. We set up a training population of 200 founders: 96 varieties from the WHEAT data, 104 obtained from the former via random mating without selfing using the **HaploSim** R package [50]. **HaploSim** assumes that markers are allocated at regular intervals across the genome, we allocated them uniformly in 21 chromosomes (wheat varieties in the WHEAT data are allohexaploid, with 2*n* = 6*x* = 42) to obtain roughly the desired amount of recombination and to preserve the linkage disequilibrium patterns as much as possible.
2. We generate phenotypes by selecting causal variants at random among markers with minor allele frequency > 5% and assigning them normally-distributed additive effects with mean zero. Noise is likewise normally distributed with mean zero and standard deviation 1, and the standard deviation of the additive effects is set such that *h*^2^ ≈ 0.55. We choose this value as the mid-point of a range of heritabilities, [0.40, 0.70], we consider to be of interest.
3. We fit a genomic prediction model on the whole training population.
4. For 100 times, we perform a sequence of 10 rounds of selection. In each round:
  - we generate the marker profiles of 200 progeny via random mating, again without selfing;
  - we generate the phenotypes for the progeny as in step 2;
  - we compute the ${\hat{F}}_{\text{ST}}$ between the training population and the progeny generated in 4a;
  - we use the marker profiles from step 4a and the genomic prediction model from 3 to obtain predicted values for the phenotypes, which are then used together with those from step 4b to compute predictive correlation;
  - we select the 20 individuals with the largest phenotypes as the parents of the next round of selection.
5. We compute the average predictive correlation $\bar{\rho }$ and the average ${\hat{F}}_{\text{ST}}$ for each round of selection, which are used as reference points to assess how well the results of the genomic selection simulation are predicted by the decay curve.
6. We estimate the decay curve $\left({\hat{F}}_{\text{ST}}^{\left(m\right)},{\hat{\rho }}_{D}^{\left(m\right)}\right)$ and its linear approximation ${\hat{\rho }}_{L}$ from the training population, and we compare it with the average $\left({\hat{F}}_{\text{ST}},\bar{\rho }\right)$ reference points from step 5.

We then repeat this simulation after adding the varieties available at the end of the second round of selection to the training population while considering the scenario with 200 and 1000 causal variants. The size of the training population is thus increased to 800 varieties, allowing us to explore the effects of a larger sample size and of considering new varieties from the breeding program to update the genomic prediction models when their predictive accuracy is no longer acceptable. In the following, we refer to this second population as the “augmented population” as opposed to the “original population” including only the 200 varieties described in steps 1 and 2 above.

#### Cross-population prediction

We explore cross-population predictions using the HUMAN data and simulated phenotypes. Similarly to the above, we pick 5, 20, 100, 2000, 10000 and 50000 causal variants at random among those with minor allele frequency > 5% and we assign them normally-distributed effects such that *h*^2^ ≈ 0.55. The same effect sizes are used for all populations. We then use individuals from Asia as the training population to estimate the decay curves. Those from other continents are the target populations for which we are assessing predictive accuracy, and we compute their ${\hat{F}}_{\text{ST}}$ and the corresponding predictive correlations ${\hat{\rho }}_{P}$. We use the $\left({\hat{F}}_{\text{ST}},{\hat{\rho }}_{P}\right)$ points as terms of comparison to assess the quality of the curve, which should be close to them or at least cross the respective 95% confidence intervals.

### Real-World Data Analyses

Finally, we estimate the decay curves for some of the phenotypes available in the WHEAT and MICE data. For both data sets we also produce and average 40 values of ${\hat{\rho }}_{\text{CV}}$ using hold-out cross-validation. In hold-out cross-validation we repeatedly split the data at random into training and target subsamples whose sizes are fixed to be the same as those arising from clustering in step 1 of the decay curve estimation. Then we fit an elastic net model on the training subsamples and predict the phenotypes in the target subsamples to estimates ${\hat{\rho }}_{\text{CV}}$. Ideally, the decay curve should cross the area in which the $\left({\hat{F}}_{\text{ST}},{\hat{\rho }}_{\text{CV}}\right)$ points cluster.

#### WHEAT data

For the WHEAT data, we construct decay curves for grain yield, height, flowering time and grain protein content using the French wheat varieties as the training population. UK and German varieties are the target populations, for which we estimate $\left({\hat{F}}_{\text{ST}},{\hat{\rho }}_{P}\right)$. Furthermore, we also construct a second decay curve for yield using the varieties registered before 1990 as the training population, as in [4]. Varieties registered between 1990 and 1999, and those registered after 2000, are used as target populations.

#### MICE data

For the MICE data, we construct decay curves for both growth rate and weight using each of the F005, F008, F010 and F016 inbred families in turn as the training population; the remaining families are used as target populations.

## Results

### General Considerations

**Fig 1:**
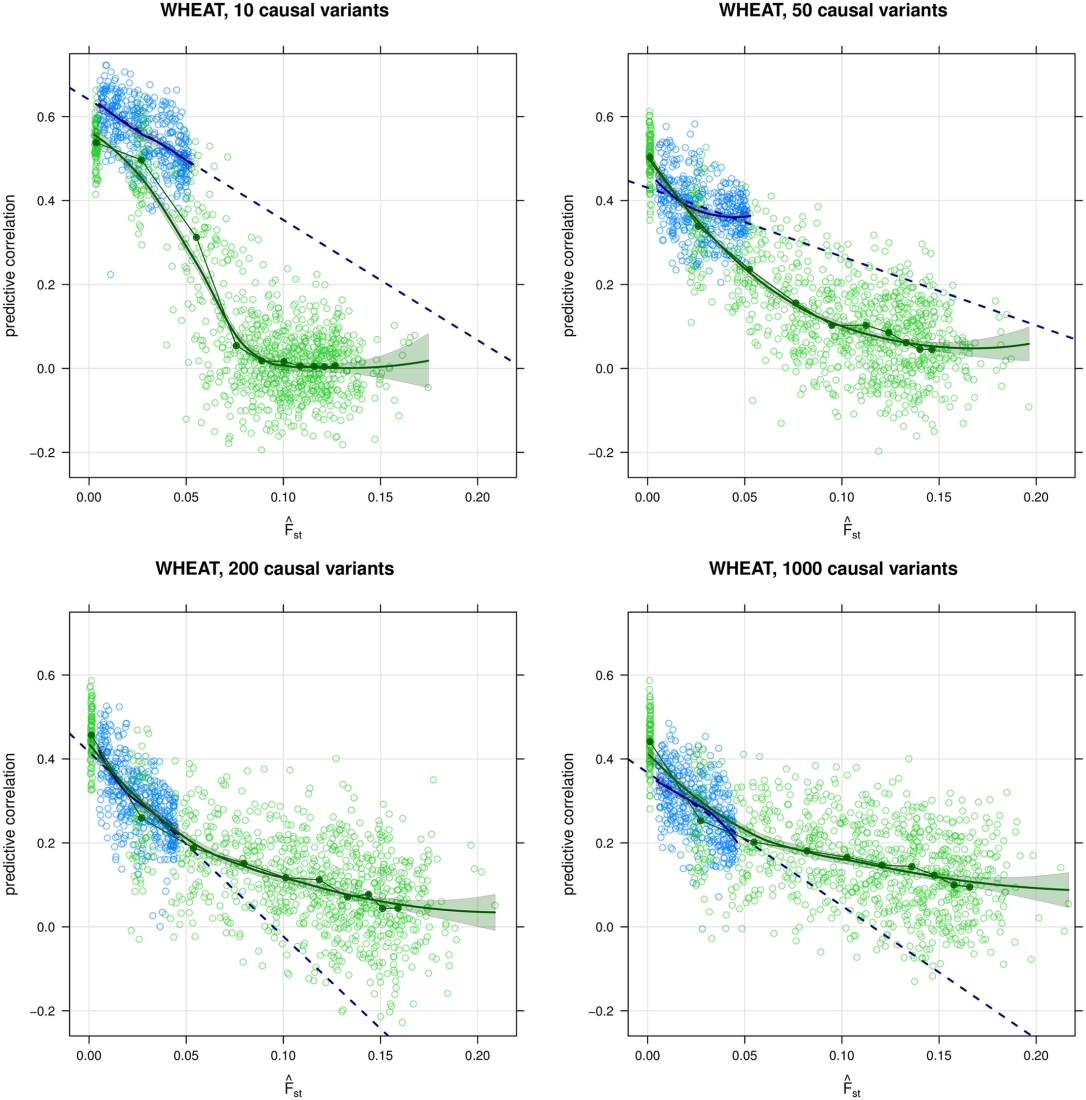
Simulation of a 10-generation breeding program using 200 varieties from the WHEAT data. Simulation of a 10-generation breeding program developed using 200 varieties generated from 2002–2007 WHEAT data with 10 (top left), 50 (top right), 200 (bottom left) and 1000 (bottom right) causal variants. The decay curves, the ${\hat{\rho }}_{D}^{\left(0\right)}$ and the ${\hat{\rho }}_{D}^{\left(m\right)}$ are in blue, and their linear interpolation (${\hat{\rho }}_{L}$) is shown as a dashed blue line. The open green circles are predictive correlations for the simulated populations, and the green solid points are the mean $\left({\hat{F}}_{\text{ST}},\bar{\rho }\right)$ for each generation.

**Fig 2:**
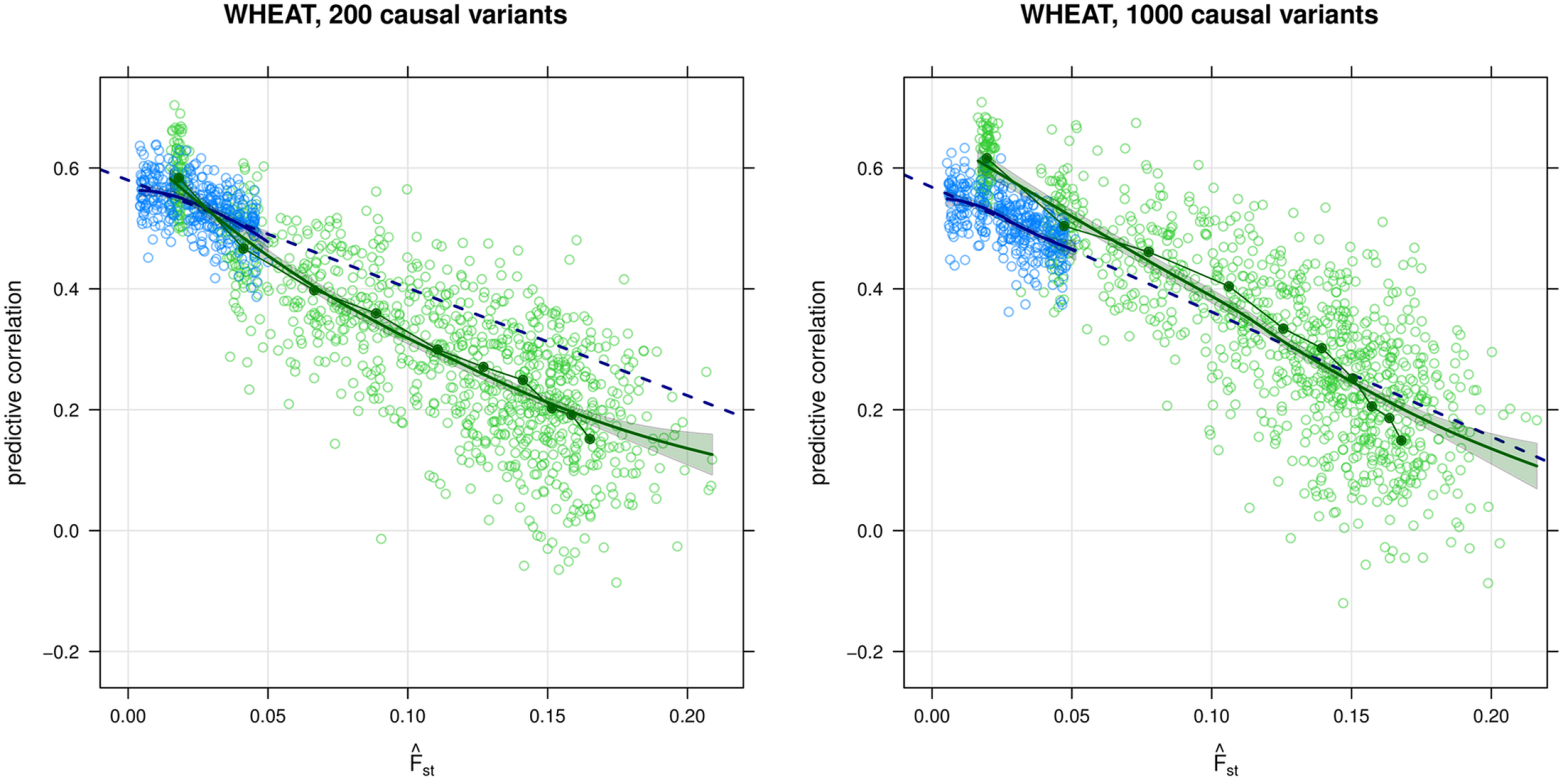
Simulation of a 10-generation breeding program with a training population augmented to 800 varieties, after two rounds of selection. Simulation of a 10-generation breeding program with an updated genomic prediction model. The updated model is fitted on the 800 varieties available after the second round of selection in the simulations for 200 (left) and 1000 (right) causal variants in Fig 1. Formatting is the same as in Fig 1.

**Fig 3:**
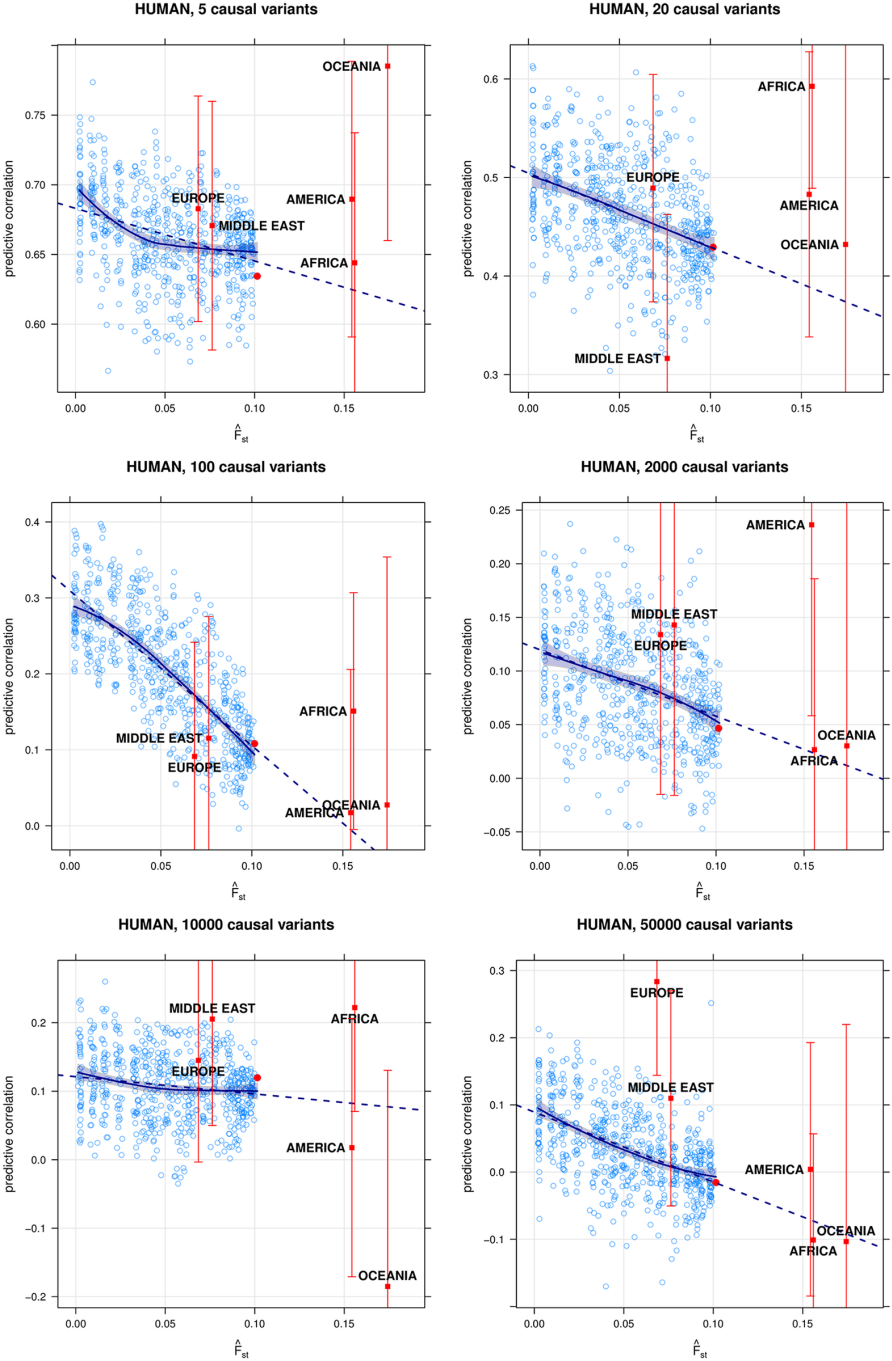
Simulation of quantitative traits from the HUMAN data. Simulation of quantitative traits with 5 (top left), 20 (top right), 100 (middle left), 2000 (middle right), 10000 (bottom left) and 50000 (bottom right) causal variants from the Asian individuals in the HUMAN data. The blue circles are the ${\hat{\rho }}_{D}^{\left(m\right)}$ used to build the curve, and the red point is ${\hat{\rho }}_{D}^{\left(0\right)}$. The blue line is the mean decay trend, with a shaded 95% confidence interval, and the dashed blue line is the linear interpolation provided by the ${\hat{\rho }}_{L}$. The red squares labelled EUROPE, MIDDLE EAST, AMERICA, AFRICA and OCEANIA correspond to the ${\hat{\rho }}_{P}$ for the individuals from those continents, and the red brackets are the respective 95% confidence intervals.

The decay curves from the simulations are shown in Figs 1, 2 and 3, and the corresponding predictive correlations are reported in Tables 1 and 2 and S1 Text. The predictive correlations for the WHEAT and MICE data sets are reported in Table 2, and the decay curves are shown in Figs 1, 2 and 3 and S1 Text. A summary of the different predictive correlations defined in the Methods and discussed here is provided in Table 1.

**Table 1:** Summary of the predictive correlations defined in the Methods.

${\hat{\rho }}_{\text{CV}}$	Predictive correlation computed on the whole training population by hold-out cross-validation with random splits.
${\hat{\rho }}_{D}^{\left(m\right)}$	Predictive correlation for a target subsample computed from a genomic prediction model fitted on the corresponding training subsample after swapping *m* individuals between the two. Used to construct the decay curve via LOESS together with the corresponding ${\hat{F}}_{\text{ST}}^{\left(m\right)}$. The subsamples are created from the training population via clustering to be minimally related.
${\hat{\rho }}_{D}$	Predictive correlation estimated by the decay curve at a given ${\hat{F}}_{\text{ST}}$.
${\hat{\rho }}_{L}$	Linear approximation to the decay curve computed by regressing the ${\hat{\rho }}_{D}^{\left(m\right)}$ against the associated ${\hat{F}}_{\text{ST}}^{\left(m\right)}$.
${\hat{\rho }}_{P}$	Predictive correlation for a target population computed by fitting a genomic prediction model on the whole training population, used as a reference point in assessing the decay curve.
$\bar{\rho }$	Mean predictive correlation for a generation in the genomic selection simulation, computed from a genomic prediction model fitted on the founders.

**Table 2:** Predictive correlations for the analyses shown in Figs B.1, B.2 and B.3 in S1 Text.

Trait	Training Population	Target Population	*n*_TR_	*n*_*TA*_	${\hat{F}}_{\text{ST}}^{\left(0\right)}$	${\hat{\rho }}_{P}$	${\hat{\rho }}_{D}$	${\hat{\rho }}_{L}$
WHEAT, Yield	France	UK	132	70	0.031	0.55	0.60	0.58
	France	Germany	132	70	0.042	0.56	0.56	0.51
WHEAT, Height	France	UK	132	70	0.031	0.57	0.63	0.58
	France	Germany	132	70	0.042	0.60	0.55	0.54
WHEAT, Flowering time	France	UK	132	70	0.031	0.36	0.70	0.70
	France	Germany	132	70	0.042	0.23	0.67	0.68
WHEAT, Grain protein content	France	UK	132	70	0.031	0.59	0.54	0.51
	France	Germany	132	70	0.042	0.47	0.46	0.45
MICE, Weight	F005	F008	155	132	0.065	0.14	0.18	0.21
	F005	F010	155	132	0.062	0.17	0.20	0.21
	F005	F016	155	132	0.061	0.15	0.20	0.22
	F008	F005	203	90*	0.066	0.24	-	0.30
	F008	F010	203	90*	0.063	0.21	-	0.31
	F008	F016	203	90*	0.056	0.16	-	0.34
	F010	F005	241	90*	0.063	0.39	-	0.52
	F010	F008	241	90*	0.062	0.22	-	0.52
	F010	F016	241	90*	0.067	0.18	-	0.52
	F016	F005	238	70*	0.063	0.34	0.29	0.35
	F016	F008	238	70*	0.057	0.07	0.32	0.35
	F016	F010	238	70*	0.069	0.27	-	0.30
MICE, Growth rate	F005	F008	207	80*	0.065	0.10	0.19	0.20
	F005	F010	207	80*	0.062	0.02	0.19	0.20
	F005	F016	207	80*	0.061	0.05	0.20	0.20
	F008	F005	199	90*	0.066	0.18	-	0.19
	F008	F010	199	90*	0.063	0.08	-	0.19
	F008	F016	199	90*	0.056	0.05	-	0.21
	F010	F005	237	90*	0.063	0.03	0.12	0.13
	F010	F008	237	90*	0.062	0.07	0.12	0.14
	F010	F016	237	90*	0.067	0.01	-	0.11
	F016	F005	219	90*	0.063	0.00	-	0.05
	F016	F008	219	90*	0.057	0.06	0.07	0.06
	F016	F010	219	90*	0.069	0.04	-	0.03

${\hat{\rho }}_{P}$ is the predictive correlation for the target population from the full training population. ${\hat{\rho }}_{D}$ is the decay curve estimate of ${\hat{\rho }}_{P}$, and is only available if the target population falls within the span of the decay curve. ${\hat{\rho }}_{L}$ is the corresponding estimate from the linear extrapolation. *n*_TR_ is the size of the training subsamples and *n*_*TA*_ is the size of the target subsamples; those marked with an asterisk have been reduced to increase *n*_TR_.

In all the simulations and the real-world data analyses the ${\hat{\rho }}_{D}$ from the decay curve is close to the linear interpolation ${\hat{\rho }}_{L}$; considering all the reference populations in Table 2 and the generation means in Tables A.1 and A.2 in S1 Text, $|{\hat{\rho }}_{D}-{\hat{\rho }}_{L}|\ll 0.02$ 41 times out of 47 (87%). Both estimates of predictive correlation are close to the respective reference values $\bar{\rho }$ and ${\hat{\rho }}_{P}$; the difference (in absolute value) is ≪ 0.05 39 times (41%) and ≪ 0.10 69 times (73%) out of 94. The proportion of small differences increases when considering only target populations that fall within the span of the decay curve: 23 out of 44 (52%) are ≪ 0.05 and 38 are ≪ 0.10 (84%). This is expected because the decay curve is already an extrapolation from the training population, so extending it further with the linear interpolation ${\hat{\rho }}_{L}$ reduces its precision. Regressing ${\left({\hat{\rho }}_{D}^{\left(m\right)}\right)}^{2}$ against the ${\left({\hat{F}}_{\text{ST}}^{\left(m\right)}\right)}^{2}$ does not produce a stronger linear relationship than that represented by ${\hat{\rho }}_{L}$ (*p* = 0.784, see Section D in S1 Text).

The range of the predictive correlations ${\hat{\rho }}_{D}^{\left(m\right)}$ around the decay curves varies between 0.05 and 0.10, and it is constant over the range of observed ${\hat{F}}_{\text{ST}}$ for each curve. It does not appear to be related to either the size of the training subsample or the number of causal variants. This is apparent in particular from the genomic selection simulation, in which both are jointly set to different combinations of values. Similarly, there seems to be no relationship between the spread and the magnitude of the predictive correlations (${\hat{\rho }}_{D}^{\left(m\right)}\in \left[0,0.75\right]$). This amount of variability is comparable to that of other studies (e.g., the range of the ${\hat{\rho }}_{D}^{\left(m\right)}$ is smaller than that in the cross-validated correlations in [32]) once we take into account that the $\left({\hat{F}}_{\text{ST}}^{\left(m\right)},{\hat{\rho }}_{D}^{\left(m\right)}\right)$ are individual predictions and are not averaged over multiple repetitions. Furthermore, subsampling further reduces the size of the training subpopulations; and fitting the elastic net requires a search over a grid of values for its two tuning parameters, which may get stuck in local optima.

### Real-World Data Analyses

Several interesting points arise from the analysis of the real phenotypes in the WHEAT and MICE data, shown in Table 2 and in Figs B.1, B.2 and B.3 in S1 Text. Firstly, cross-validation always produces pairs of subsamples with ${\hat{F}}_{\text{ST}}⩽0.01$ and high ${\hat{\rho }}_{\text{CV}}$ that are located at the left end of the decay curve. The average ${\hat{F}}_{\text{ST}}$ is 0.006 for the WHEAT data and 0.001 for the MICE data, and the difference between the average ${\hat{\rho }}_{\text{CV}}$ and the corresponding ${\hat{\rho }}_{D}$ is ≪ 0.02 10 times out of 12 (83%, see Table B.4 in S1 Text). The spread of the ${\hat{\rho }}_{\text{CV}}$ is also similar to that of the ${\hat{\rho }}_{D}^{\left(m\right)}$. Secondly, we note that in the WHEAT data all decay curves but that for flowering time cross the 95% confidence intervals for the cross-country predictive correlations ${\hat{\rho }}_{P}$ for Germany and UK reported in [4]. Even in the MICE data, in which all families are near the end or beyond the reach of the decay curves, the latter (or their linear approximations) cross the 95% confidence intervals for the ${\hat{\rho }}_{P}$ 18 times out of 24 (75%). However, we also note that those intervals are wide due to the limited sizes of those populations.

Furthermore, the decay curves for the phenotypes in the WHEAT data confirm two additional considerations originally made in [4]. Firstly, [4] noted that the distribution of the Ppd-D1a gene, which is a major driver of this flowering time, varies substantially with the country of registration and thus cross-country predictions are not reliable. Fig B.1 in S1 Text shows that the decay curve vastly overestimates the predictive correlation for both Germany and the UK. Splitting the WHEAT data in two halves that contain equal proportions of both alleles of Ppd-D1a and that are genetically closer overall (${\hat{F}}_{\text{ST}}=0.04$), we obtain a decay curve that fits the predictive correlations reported in the original paper (${\hat{\rho }}_{D}=0.77$, ${\hat{\rho }}_{P}=0.79$). Secondly, we also split the data according to their year of registration and use the oldest varieties (pre-1990) as a training sample for predicting yield. Again the decay curve crosses the 95% confidence intervals for the predictive correlations reported in [4] and the correlations themselves are within 0.05 of the average ${\hat{\rho }}_{D}$ from the decay curve both for 1990-1999 (${\hat{F}}_{\text{ST}}=0.028$, ${\hat{\rho }}_{D}=0.44$, ${\hat{\rho }}_{P}=0.40$) and post-2000 (${\hat{F}}_{\text{ST}}=0.033$, ${\hat{\rho }}_{D}=0.44$, ${\hat{\rho }}_{P}=0.42$) varieties.

### Simulation Studies

The decay curves from the genomic selection simulation on the original training population (200 varieties), shown in blue in Fig 1, span two rounds of selection and three generations. When considering 200 or 1000 causal variants, the curve overlaps the mean behaviour of the simulated data points (shown in green) almost perfectly: the difference between the generation means $\bar{\rho }$ and the decay curve is ⩽ 0.06 for the first three generations, with the exception of the first generation in the simulation with 1000 variants ($|\bar{\rho }-{\hat{\rho }}_{D}|=0.09$). As the number of causal variants decreases (50, 10), the decay curve increasingly overestimates $\bar{\rho }$, although the difference remains ⩽0.10 for the first two generations; and both show a slower decay than the $\bar{\rho }$. This appears to be due to a few alleles of large effect becoming fixed by the selection, leading to a rapid decrease of $\bar{\rho }$ without a corresponding rapid increase in ${\hat{F}}_{\text{ST}}$.

The decay curves fitted on the augmented training populations (800 varieties, now including those available at the end of the second round of selection, Fig 2) fit the first four generations well ($|\bar{\rho }-{\hat{\rho }}_{D}|⩽0.04$ for the first two, $|\bar{\rho }-{\hat{\rho }}_{D}|⩽0.06$ for the third and the fourth). As before, the only exception is the first generation in the simulation with 1000 variants, with an absolute difference of 0.09. However, the decay curves are also able to capture the long-range decay rates through their linear approximations. When considering 200 causal variants, $|\bar{\rho }-{\hat{\rho }}_{L}|\approx 0.08$ for generations 5 to 7 and ≈0.10 for generations 8 and 9; and $|\bar{\rho }-{\hat{\rho }}_{L}|\ll 0.05$ for generations 4 to 9 when considering 1000 causal variants. This can be attributed to the increased sample size of the training population, which both improves the goodness of fit of the estimated decay curve; and makes the decay rate of the $\bar{\rho }$ closer to linear, thus making it possible for the ${\hat{\rho }}_{L}$ to approximate it well over a large range of *F*_ST_ values. To investigate this phenomenon, we gradually increased the initial training population to 4000 varieties through random mating and we observed that for such a large sample size $\bar{\rho }$ indeed decreases linearly as a function of *F*_ST_. We conjecture that this is due to a combination of the higher values observed for $\bar{\rho }$ and their slower rate of decay, which prevents the latter from gradually decreasing as $\bar{\rho }$ is still far from zero after 10 generations. In addition, we note that increasing the number of causal variants has a similar effect; with 200 and 1000 causal variants $\bar{\rho }$ indeed decreases with an approximately linear trend, which is not the case with 10 and 50 causal variants.

The cross-population prediction simulation based on the HUMAN data (Fig 3) generated results consistent with those above. As before, the number of causal variants appears to influence the behaviour of the decay curve: while the ${\hat{\rho }}_{D}^{\left(m\right)}$ decrease linearly for 20, 100 and 2000 casual variants, they converge to 0.65 for 5 causal variants. However, unlike in the genomic selection simulation, the quality of the estimated decay curve does not appear to degrade as the number of causal variants decreases. This difference may depend on the lack of a systematic selection pressure in the current simulation, which made the decay curve overestimate predictive correlation when considering 10 variants in the previous simulation. Finally, as in the analysis of the MICE data, the linear approximation ${\hat{\rho }}_{L}$ to the decay curve provides a way to extend the reach of the decay curve to estimate predictive correlations ${\hat{\rho }}_{P}$ for distantly related populations (AMERICA, AFRICA, OCEANIA). Again we observe some loss in precision (see Table 2), but the extension still crosses the 95% confidence intervals of those ${\hat{\rho }}_{P}$ 14 times out of 18 (78%).

## Discussion

Being able to assess the predictive accuracy is important in many applications, and will assist in the development of new models and in the choice of training populations. A number of papers have discussed various aspects of the relationship between training and target populations in genomic prediction, and of characterising predictive accuracy given some combination of genotypes and pedigree information. For instance, [51] discusses how to choose which individuals to include in the training population to maximise prediction accuracy for a given target population using the coefficient of determination. [52] separates the contributions of linkage disequilibrium, co-segregation and additive genetic relationships to predictive accuracy, which can help in setting expectations about the possible performance of prediction. [53] and [22] link predictive accuracy to kinship in a simulation study of dairy cattle breeding; and [54] investigates the impact of population size, population structure and replication in a simulated biparental maize populations. The approach we take in this paper is different in a few, important ways. Firstly, we choose to avoid the parametric assumptions underlying GBLUP and the corresponding approximations based on Henderson’s equations that provide closed-form results on predictive accuracy in the literature. It has been noted in our previous work [31] and in the literature (e.g. [32]) that in some settings GBLUP may not be competitive for genomic prediction; hence we prefer to use models with better predictive accuracy such as the elastic net for which the parametric assumptions do not hold. Our model-agnostic approach is beneficial also because decay curves can then be constructed for current and future competitive models, since the only requirement of our approach is that they must be able to produce an estimate of predictive correlation. Secondly, we demonstrate that the decay curves estimated with the proposed approach are accurate in different settings and on human, plant and animal real-world data sets. This complements previous work that often used synthetic genotypes and analysed predictive accuracy in a single domain, such as forward simulation studies on dairy cattle data. Finally, we recognise that the target population whose phenotypes we would like to predict may not be available or even known when training the model. In plant and animal selection programs, one or more future rounds of crossings may not yet have been performed; in human genetics, prediction may be required into different demographic groups for which no training data are available. Therefore, we are often limited to extrapolating a ${\hat{\rho }}_{D}$ to estimate the ${\hat{\rho }}_{P}$ we would observe if the target population were available. Prior information on ${\hat{F}}_{\text{ST}}$ values is available for many species such as humans [39, 43]; and can be used to extract the corresponding ${\hat{\rho }}_{D}$ from a decay curve.

We observe that the decay rate of ${\hat{\rho }}_{D}$ is approximately linear in ${\hat{F}}_{\text{ST}}$ for most of the curves, suggesting that regressing the ${\hat{\rho }}_{D}^{\left(m\right)}$ against the ${\hat{F}}_{\text{ST}}^{\left(m\right)}$ is a viable estimation approach. This has the advantage of being computationally cheaper than producing a smooth curve with LOESS since it requires fewer $\left({\hat{F}}_{\text{ST}}^{\left(m\right)},{\hat{\rho }}_{D}^{\left(m\right)}\right)$ points and thus fewer genomic prediction models to be fitted. In fact, if we assume that the decay rate is linear we could also estimate it as the slope of the line passing through $\left({\hat{F}}_{\text{ST}}\approx 0,{\hat{\rho }}_{\text{CV}}\right)$ and $\left({\hat{F}}_{\text{ST}}^{\left(m\right)},{\hat{\rho }}_{D}^{\left(m\right)}\right)$ for a single, small value of *m*. It should be noted, however, that several factors can cause departures from linearity, including the number of causal variants underlying the trait, the use of small training populations and the confounding effect of exogenous factors. In the case of the MICE data, for instance, predictions may be influenced by cage effects; in the case of the WHEAT data, environmental and seasonal effects might not be perfectly captured and removed by the trials’ experimental design. We also note that the decay curves for traits with small heritabilities will almost never be linear, because ${\hat{\rho }}_{D}$ converges asymptotically to zero. Unlike the results reported in [22], we do not find a statistically significant difference between the strength of the linear relationship between ${\hat{\rho }}_{D}$ and ${\hat{F}}_{\text{ST}}$ and that between the respective squares. There may be several reasons for this discrepancy; the simulation study in [22] was markedly different from the analyses presented in this paper, since it used simulated genotypes to generate the population structure typical of dairy cattle and since it used GBLUP as a genomic prediction model.

We also observe that when ${\hat{F}}_{\text{ST}}^{\left(m\right)}\approx 0$, both ${\hat{\rho }}_{D}^{\left(m\right)}$ and ${\hat{\rho }}_{L}$ are, as expected, similar to the ${\hat{\rho }}_{\text{CV}}$ obtained by applying cross-validation to the training populations selected from the WHEAT and MICE data. This suggests that indeed ${\hat{\rho }}_{\text{CV}}$ is an accurate measure of predictive accuracy only when the target individuals for prediction are drawn from the same population as the training sample, as previously argued by [14] and [19], among others.

Some limitations of the proposed approach are also apparent from the results presented in the previous section. The most important of these limitations appears to be that in the context of a breeding program the performance of the decay curve depends on the polygenic nature of the trait being predicted, as we can see by comparing the panels in Fig 1. This can be explained by the fact that causal variants underlying less polygenic, highly and moderately heritable traits will necessarily have some individually large effects. As each of those variants approaches fixation due to selection pressure, allele frequencies in key areas of the genome will depart from those in the training population and the accuracy of any genomic prediction model will rapidly decrease [21]. However, these selection effects are genomically local and so have little impact on ${\hat{F}}_{\text{ST}}$. A similar effect has been observed for flowering time in the WHEAT data. [4] notes that the Ppd-D1a gene is a major driver of early flowering, but it is nearly monomorphic in one allele in French wheat varieties and nearly monomorphic in the other allele in Germany and the UK. As a result, even though the ${\hat{F}}_{\text{ST}}$ for those countries are as small as 0.031 and 0.042, ${\hat{\rho }}_{D}$ widely overestimates ${\hat{\rho }}_{P}$ in both cases. A possible solution would be to compute ${\hat{F}}_{\text{ST}}$ only on the relevant regions of the genome or, if their precise location is unknown, on the relevant chromosomes; or to weight ${\hat{F}}_{\text{ST}}$ to promote genomic regions of interest.

On the other hand, in the case of more polygenic traits a larger portion of the genome will be in linkage disequilibrium with at least one causal variant, and their effects will be individually small. Therefore, ${\hat{F}}_{\text{ST}}$ will increase more quickly in response to selection pressure and changes in predictive accuracy will be smoother, thus allowing ${\hat{\rho }}_{D}$ to track them more easily. Indeed, in the WHEAT data the genomic prediction model for flowering time has a much smaller number of non-zero coefficients (28) compared to yield (91), height (286) and grain protein content (121). Similarly, in the MICE data the model fitted on F010 to predict weight has only 168 non-zero coefficients while others range from 212 to 1169 non-zero coefficients. By contrast, all models fitted for predicting weight, which correspond to curves that well approximate other families’ ${\hat{\rho }}_{P}$, have between 1128 and 2288 non-zero coefficients.

The simulation on the HUMAN data suggests different considerations apply to outbred species. Having some large-effect causal variants does not necessarily result in low quality decay curves; on the contrary, if we assume that the trait is controlled by the same causal variants in the training and target populations it is possible to have a good level of agreement between the ${\hat{\rho }}_{D}$ and the ${\hat{\rho }}_{P}$. Intuitively, we expect strong effects to carry well across populations and thus ${\hat{\rho }}_{D}$ does not decrease beyond a certain *F*_ST_. However, this will mean that the curves will not be linear and ${\hat{\rho }}_{L}$ will underestimate ${\hat{\rho }}_{P}$ (see Fig 3, top left panel). We also note that effect sizes are the same in all the populations, which may make our estimates of predictive accuracy optimistic.

Another important consideration is that since the decay curve is extrapolated from the training population, its precision decreases as *F*_ST_ increases, as can be seen from both simulations and by comparing the WHEAT and MICE data. Predictions will be poor in practice if the target and the training populations are too genetically distinct; an example are rice subspecies [17], which have been subject to intensive inbreeding. The trait to be predicted must have a common genetic basis across training and target populations. However, the availability of denser genomic data and of larger samples may improve both predictive accuracy and the precision of the decay curve for large *F*_ST_. Furthermore, the range of the decay curve in terms of *F*_ST_ depends on the amount of genetic variability present in the training population; the more homogeneous it is, the more unlikely that *k*-means clustering will be able to split it in two subsets with high ${\hat{F}}_{\text{ST}}^{\left(0\right)}$. One solution is to assume the decay is linear and use ${\hat{\rho }}_{L}$ instead of ${\hat{\rho }}_{D}$ to estimate ${\hat{\rho }}_{P}$; but as we noted above this is only possible if ${\hat{\rho }}_{P}\gg 0$. If ${\hat{\rho }}_{P}\approx 0$, the decay curve estimated with LOESS from ${\hat{\rho }}_{D}$ can converge asymptotically to zero as ${\hat{F}}_{\text{ST}}$ increases; but the linear regression used to estimate ${\hat{\rho }}_{L}$ will continue to decrease until ${\hat{\rho }}_{L}\ll 0$. Another possible solution is to try to increase ${\hat{F}}_{\text{ST}}$ by moving observations between the two subsets, but improvements are marginal at best and there is a risk of inflating ${\hat{\rho }}_{D}$.

Even with such limitations, estimating a decay curve for predictive correlation has many possible uses. In the context of plant and animal breeding, it is a useful tool to answer many key questions in planning genomic selection programs. Firstly, different training populations (in terms of allele frequencies, sample size, presence of different families, etc.) can be compared to choose that which results in the slowest decay rate. Secondly, the decay curve can be used to decide when genomic prediction can no longer be assumed to be accurate enough for selection purposes, and thus how often the model should be re-trained on a new set of phenotypes. Unlike genotyping costs, phenotyping costs for productivity traits have not decreased over the years. Furthermore, the rate of phenotypic improvements (i.e. selection cycle time) can be severely reduced by the need of performing progeny tests. Therefore, limiting phenotyping to once every few generations can reduce the cost and effort of running a breeding program. The presence of close ancestors in the training population suggests that decay curves are most likely reliable for this purpose, as we have shown both in the simulations and in predicting newer wheat varieties from older ones in the WHEAT data.

The other major application of decay curves is estimating the predictive accuracy of a model for target populations that, while not direct descendants of the training population, are assumed not to have strongly diverged and thus to have comparable genetic architectures. Some examples of such settings are the cross-country predictions for the WHEAT data, the cross-family predictions for the MICE data and across human populations. In human genetics, decay curves could be used to study the accuracy of predictions and help predict the success of interventions of poorly-studied populations. In plant and animal breeding, on the other hand, it is common to incorporate distantly related samples in selection programs to maintain a sufficient level of genetic variability. Decay curves can provide an indication of how accurately the phenotypes for such samples are estimated, since the model has not been trained to predict them well and they are not as closely related as the individuals in the program.

## Supporting Information

S1 TextSupplementary information on the Methods and the Results.Figures for the decay curves from the simulation studies. Relationship between ${\hat{F}}_{\text{ST}}$ and $\bar{k}$. Comparison of the linear relationships between *ρ*^2^ versus ${\hat{F}}_{\text{ST}}^{2}$ and *ρ* and ${\hat{F}}_{\text{ST}}$.(PDF)
