# Supplementary material for: Using Genetic Distance to Infer the Accuracy of Genomic Prediction
Source: PLoS Genet. 2016 Sep 2;12(9):e1006288. doi: 10.1371/journal.pgen.1006288 (PMC5010218; doi:10.1371/journal.pgen.1006288)
Supplement: S1 Text — Figures for the decay curves from the simulation studies. Relationship between F^ST and k¯. Comparison of the linear relationships between ρ2 versus F^ST2 and ρ and F^ST. (PDF) [file pgen.1006288.s001.pdf]

Supplementary Information

Contents

A Simulation Studies **2**
A.1 Breeding Program Simulation using the WHEAT data 2
A.2 Cross-Population Simulation using the HUMAN data 4

B Real-World Data Analyses **5**
B.1 WHEAT Data 5
B.2 MICE Data 6
B.3 Cross-Validation and Decay Curve in the WHEAT and MICE data 8

C Kinship and F_{ST} **9**

D Relationship between Squared Predictive Correlation and F_{ST}^2 **12**

A Simulation Studies

A.1 Breeding Program Simulation using the WHEAT data

**Causal Variants**	**Generation**	$\hat{F}_{\text{ST}}$	$\bar{\rho}$	$\hat{\rho}_{\text{D}}$	$\hat{\rho}_{\text{L}}$
10	1	0.003	0.54	0.61	0.63
10	2	0.027	0.50	0.56	0.56
10	3	0.055	0.31	0.49	0.48
50	1	0.001	0.50	0.44	0.43
50	2	0.026	0.34	0.38	0.39
50	3	0.052	0.24	0.36	0.34
200	1	0.001	0.46	0.40	0.41
200	2	0.027	0.26	0.29	0.29
200	3	0.053	0.19	0.23	0.18
1000	1	0.001	0.44	0.35	0.36
1000	2	0.027	0.25	0.29	0.28
1000	3	0.055	0.20	0.18	0.19

Table A.1. Predictive correlations for the simulations shown in Figure 1 in the paper; the training population for the genomic prediction model is composed by 200 varieties from 2002–2007 WHEAT data. $\bar{\rho}$ is the average predictive correlation for a given generation, training population size and number of causal variants; and $\hat{F}_{\text{ST}}$ is the corresponding average F_{ST}. $\hat{\rho}_{\text{D}}$ is the decay curve estimate of $\bar{\rho}$, and is only available if the generation average falls within the span of the decay curve. $\hat{\rho}_{\text{L}}$ is the corresponding estimate from the linear extrapolation.

**Causal Variants**	**Generation**	$\hat{F}_{\mathrm{ST}}$	$\bar{\rho}$	$\hat{\rho}_{\mathrm{D}}$	$\hat{\rho}_{\mathrm{L}}$
200	1	0.018	0.58	0.55	0.55
200	2	0.041	0.47	0.51	0.51
200	3	0.066	0.40	–	0.46
200	4	0.088	0.36	–	0.42
200	5	0.111	0.30	–	0.38
200	6	0.127	0.27	–	0.35
200	7	0.141	0.25	–	0.33
200	8	0.151	0.20	–	0.31
200	9	0.158	0.19	–	0.30
200	10	0.165	0.15	–	0.28
1000	1	0.019	0.62	0.53	0.53
1000	2	0.047	0.50	0.48	0.47
1000	3	0.077	0.46	–	0.41
1000	4	0.106	0.40	–	0.35
1000	5	0.126	0.33	–	0.31
1000	6	0.139	0.30	–	0.28
1000	7	0.150	0.25	–	0.26
1000	8	0.157	0.20	–	0.24
1000	9	0.164	0.19	–	0.23
1000	10	0.168	0.15	–	0.22

Table A.2. Predictive correlations for the simulations shown in Figure 2 in the paper; the training population for the genomic prediction model is composed by the 800 varieties available after the second round of selection in the simulation. The notation is the same as in Table A.1.

A.2 Cross-Population Simulation using the HUMAN data

**Training Population**	**Target Population**	**Causal Variants**	$\hat{F}_{\mathrm{ST}}$	$\hat{\rho}_{\mathrm{P}}$	$\hat{\rho}_{\mathrm{D}}$	$\hat{\rho}_{\mathrm{L}}$
Asia	Europe	5	0.068	0.68	0.65	0.66
	Middle east	5	0.076	0.67	0.65	0.65
	America	5	0.154	0.69	–	0.62
	Africa	5	0.156	0.64	–	0.62
	Oceania	5	0.174	0.78	–	0.62
Asia	Europe	20	0.068	0.49	0.45	0.45
	Middle east	20	0.076	0.32	0.39	0.39
	America	20	0.154	0.48	–	0.39
	Africa	20	0.156	0.59	–	0.45
	Oceania	20	0.174	0.43	–	0.37
Asia	Europe	100	0.068	0.09	0.17	0.17
	Middle east	100	0.076	0.12	0.15	0.15
	America	100	0.154	0.02	–	0.00
	Africa	100	0.156	0.15	–	0.00
	Oceania	100	0.174	0.03	–	−0.05
Asia	Europe	2000	0.068	0.13	0.08	0.08
	Middle east	2000	0.076	0.14	0.07	0.07
	America	2000	0.154	0.24	–	0.02
	Africa	2000	0.156	0.03	–	0.02
	Oceania	2000	0.174	0.03	–	0.01
Asia	Europe	10000	0.068	0.15	0.10	0.10
	Middle east	10000	0.076	0.21	0.10	0.10
	America	10000	0.154	0.02	–	0.08
	Africa	10000	0.156	0.22	–	0.08
	Oceania	10000	0.174	−0.18	–	0.08
Asia	Europe	50000	0.068	0.28	0.02	0.02
	Middle east	50000	0.076	0.11	0.01	0.01
	America	50000	0.154	0.00	–	−0.07
	Africa	50000	0.156	−0.10	–	−0.07
	Oceania	50000	0.174	−0.10	–	−0.09

Table A.3. Predictive correlations for the simulations shown in Figure 3 in the paper. $\hat{\rho}_{\mathrm{P}}$ is the predictive correlation for the target population from the full training population. $\hat{\rho}_{\mathrm{D}}$ is the decay curve estimate of $\hat{\rho}_{\mathrm{P}}$, and is only available if the target population falls within the span of the decay curve. $\hat{\rho}_{\mathrm{L}}$ is the corresponding estimate from the linear extrapolation.

B Real-World Data Analyses

B.1 WHEAT Data

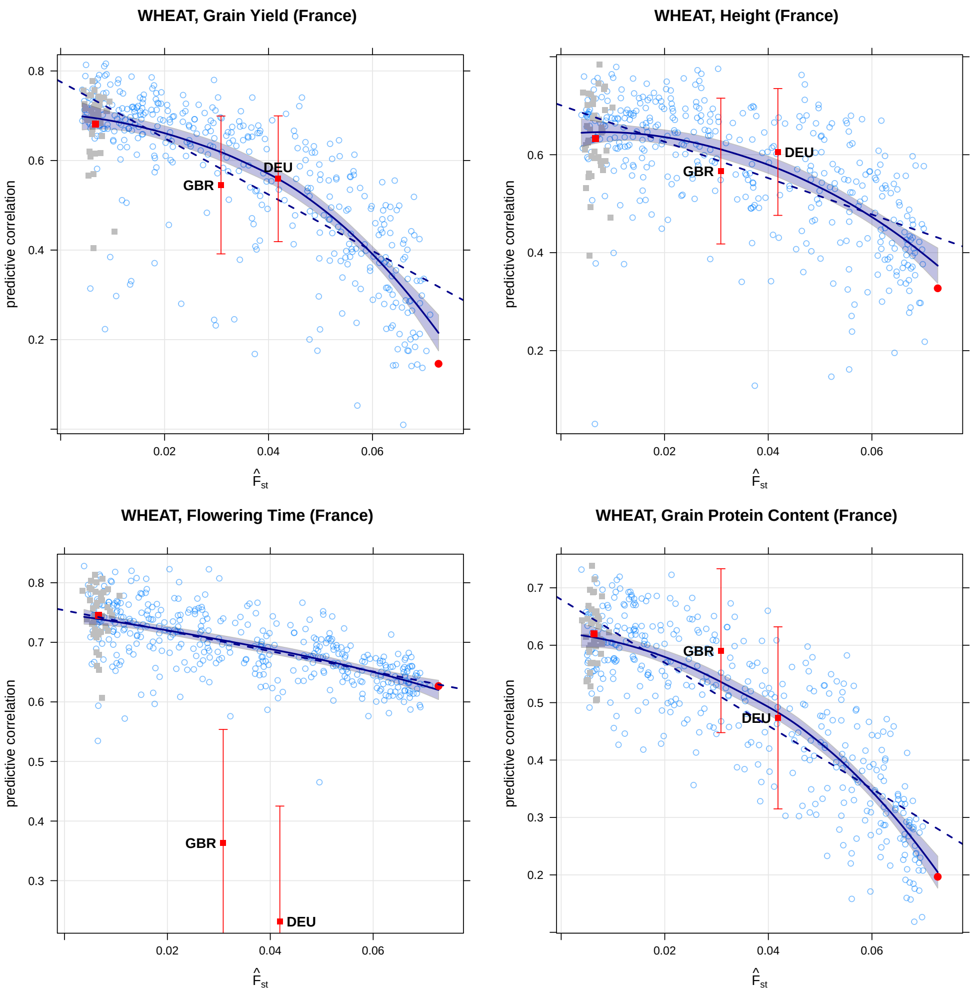

Figure B.1. Decay curves for grain yield, height, flowering time and grain protein content estimated from the French wheat varieties in the WHEAT data. The blue circles are the $\hat{\rho}_{\mathrm{D}}^{(m)}$ used to build the curve, and the red point is $\hat{\rho}_{\mathrm{D}}^{(0)}$. The blue line is the mean decay trend, with a shaded 95% confidence interval, and the dashed blue line is the linear interpolation provided by the $\hat{\rho}_{\mathrm{L}}$. Gray squares are the $\hat{\rho}_{\mathrm{CV}}$ computed using hold-out cross-validation. The red squares labelled GBR and DEU correspond to the $\hat{\rho}_{\mathrm{P}}$ for the British and German varieties, and the red brackets are the respective 95% confidence intervals.

B.2 MICE Data

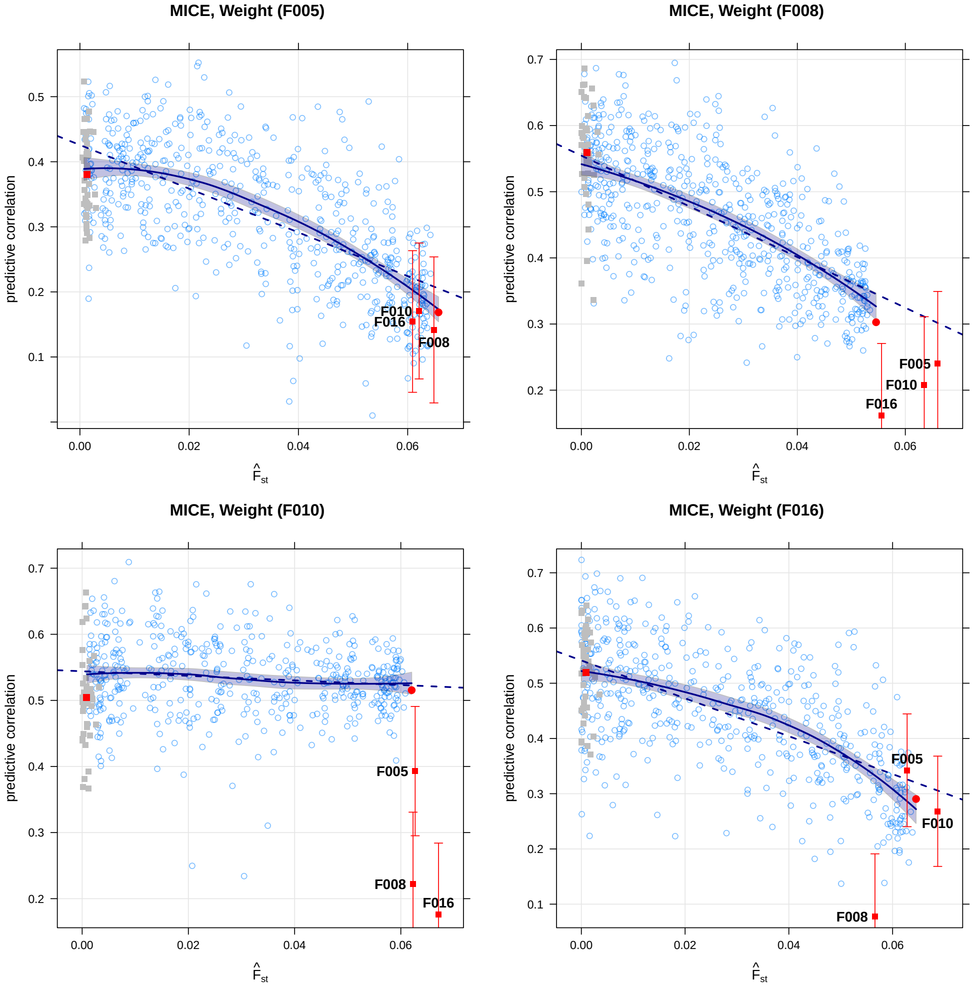

Figure B.2. Decay curves for weight estimated from the 4 largest families in the MICE data, labelled F005, F008, F010 and F016. The red squares in each panel correspond to the predictive correlations for the populations not used for estimating the decay curve; the red brackets are 95% confidence intervals. Formatting is the same as in Figure B.1.

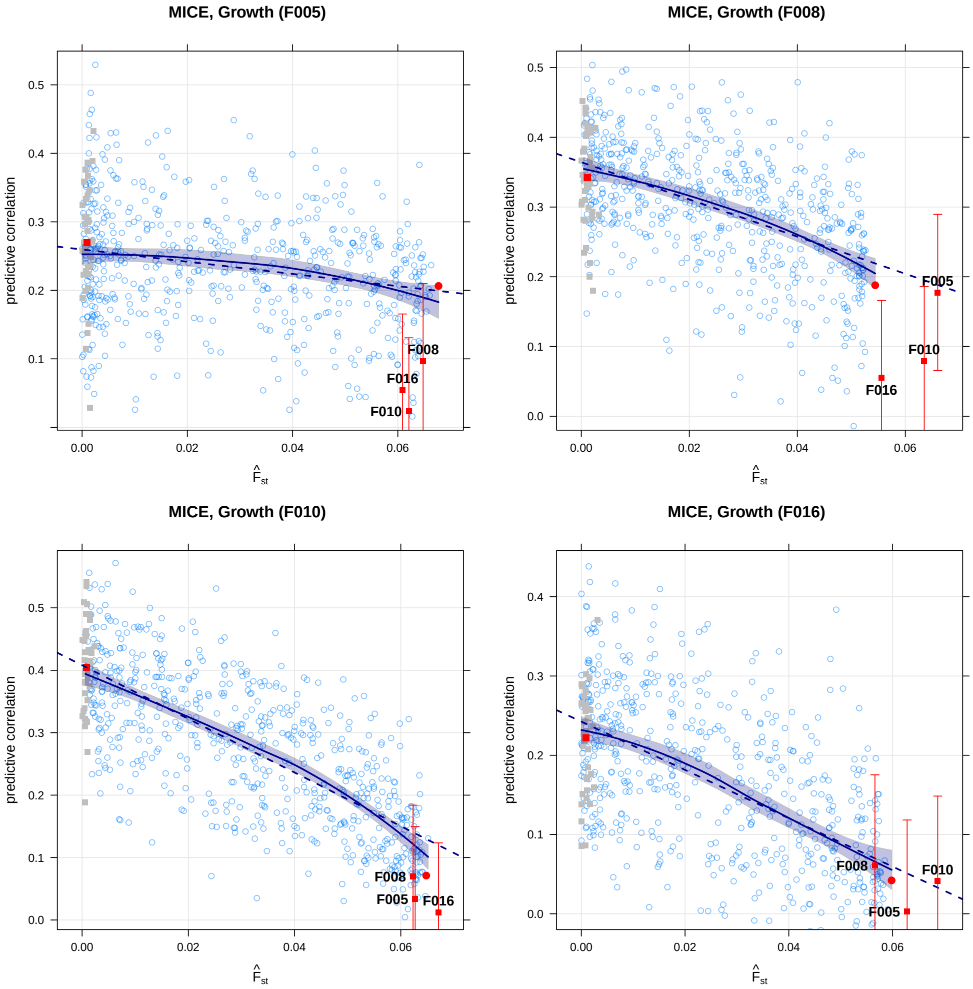

Figure B.3. Decay curves for growth rate estimated from the 4 largest families in the MICE data, labelled F005, F008, F010 and F016. The red squares in each panel correspond to the predictive correlations for the populations not used for estimating the decay curve; the red brackets are 95% confidence intervals. Formatting is the same as in Figure B.1.

B.3 Cross-Validation and Decay Curve in the WHEAT and MICE data

**Trait**	**Training Population**	$\hat{F}_{\mathrm{ST}}$	$\hat{\rho}_{\mathrm{CV}}$	$\hat{\rho}_{\mathrm{D}}$
WHEAT, Yield	France	0.006	0.68	0.68
WHEAT, Height	France	0.006	0.63	0.64
WHEAT, Flowering time	France	0.006	0.74	0.74
WHEAT, Grain protein content	France	0.006	0.62	0.61
MICE, Weight	F005	0.001	0.38	0.39
	F008	0.001	0.56	0.53
	F010	0.001	0.50	0.54
	F016	0.001	0.52	0.52
MICE, Growth rate	F005	0.001	0.27	0.25
	F008	0.001	0.34	0.35
	F010	0.001	0.40	0.38
	F016	0.001	0.22	0.23

Table B.4. Predictive correlations from the decay curves and from cross-validation for the analyses shown in Figures B.1, B.2 and B.3. $\hat{F}_{\mathrm{ST}}$ and $\hat{\rho}_{\mathrm{CV}}$ are the mean genetic distance and mean predictive correlation from the 40 runs of hold-out cross-validation; $\hat{\rho}_{\mathrm{D}}$ is the predictive correlation estimated by the decay curve at genetic distance $\hat{F}_{\mathrm{ST}}$.

C Kinship and F_{ST}

Data	Subset	ms	$\text{COR}(\hat{F}_{\text{ST}}^{(m)}, \bar{k}^{(m)})$	$log_{10}(p)$
WHEAT	France	401	−0.9894	−672.10
MICE	F005	601	−0.9982	−1467.58
MICE	F008	601	−0.9982	−1467.58
MICE	F010	601	−0.9906	−1038.57
MICE	F016	601	−0.9948	−1192.05
HUMAN	Asia	601	−0.9998	−2038.97

Table C.5. Correlation between $\hat{F}_{\text{ST}}^{(m)}$ and $\bar{k}^{(m)}$ in the data sets and training populations used in the paper. The p-values are computed using the exact t-test for the correlation coefficient [2] and adjusted for multiplicity via FDR [1].

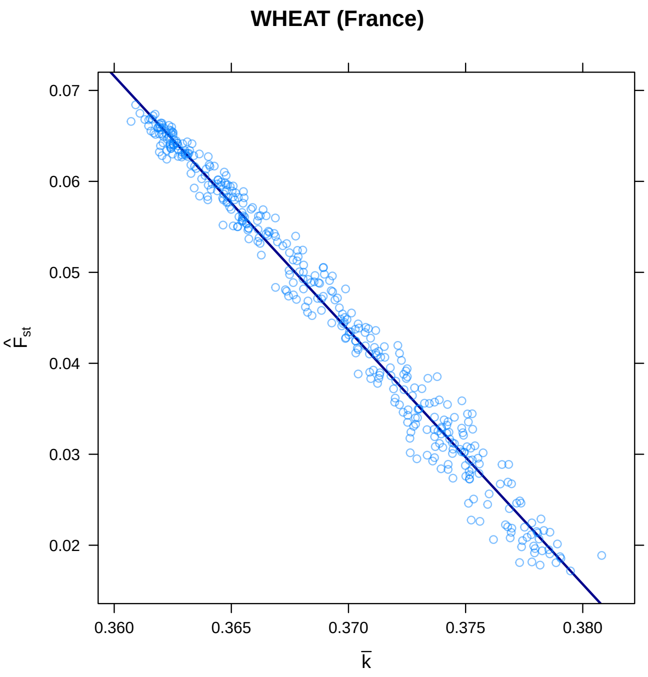

Figure C.4. $(F_{\text{ST}}^{(m)}, \bar{k}^{(m)})$ pairs generated from the French wheat varieties in the WHEAT data.

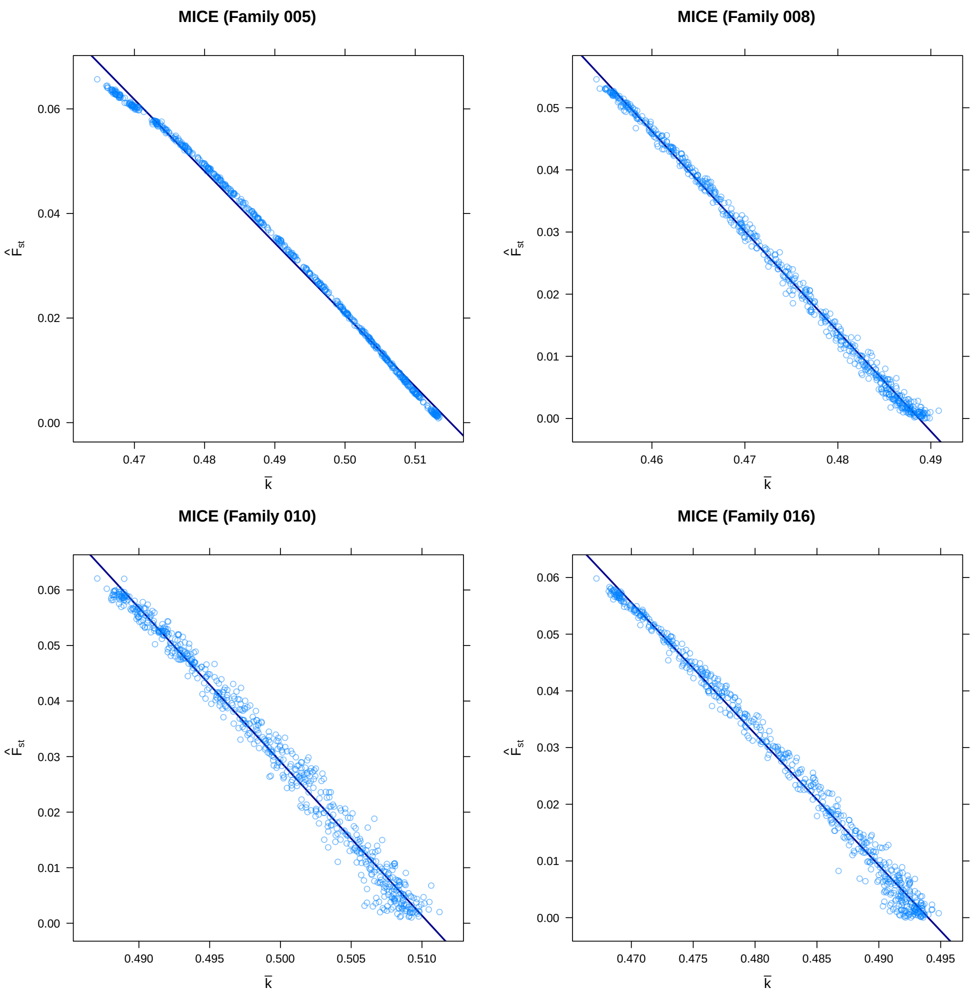

Figure C.5. $(F_{\mathrm{ST}}^{(m)}, \bar{k}^{(m)})$ pairs generated from the 4 largest families in the MICE data, labelled F005, F008, F010 and F016.

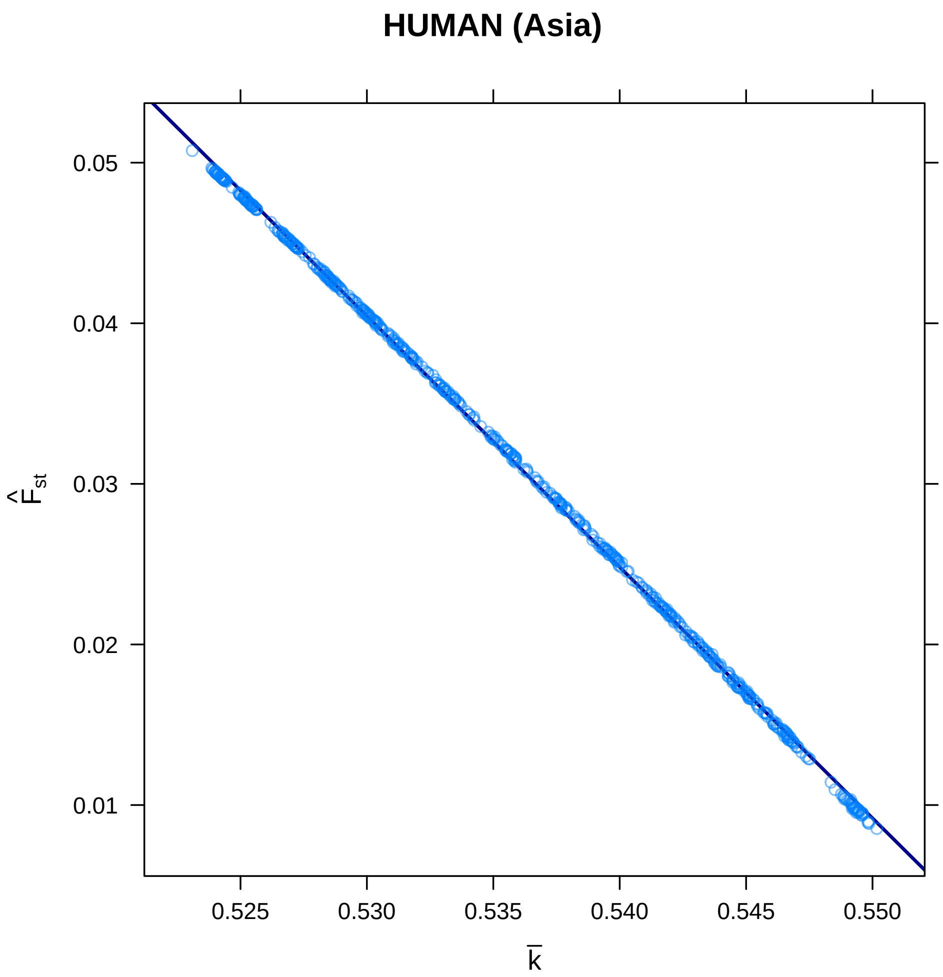

Figure C.6. $(F_{\mathrm{ST}}^{(m)}, \bar{k}^{(m)})$ pairs generated from the Asian individuals in the HUMAN data.

D Relationship between Squared Predictive Correlation and F_{ST}^2

[4] used a simulated dairy cattle population, created simulating both phenotypes and genotypes, suggested that squared predictive correlation has a stronger linear relationship with squared mean kinship than predictive correlation does with mean kinship. Predictive correlation was computed using GBLUP as a genomic prediction model.

In the context of this paper, this is equivalent to testing whether the $\left(\hat{\rho}_{\mathrm{D}}^{(m)}\right)^2$ have a stronger linear relationship with the $\left(\hat{F}_{\mathrm{ST}}^{(m)}\right)^2$ than the $\hat{\rho}_{\mathrm{D}}^{(m)}$ do with the $\hat{F}_{\mathrm{ST}}^{(m)}$; we have shown that $F_{\mathrm{ST}}^{(m)}$ and $\bar{k}^{(m)}$ are almost perfectly linearly correlated so they can be used interchangeably for this purpose. We regress the $\hat{\rho}_{\mathrm{D}}^{(m)}$ on the $\hat{F}_{\mathrm{ST}}^{(m)}$ and measure the R^2 coefficient of the resulting linear model, denoted as $\mathrm{R^2}_{\mathrm{LINEAR}}$. Similarly, we regress the $\left(\hat{\rho}_{\mathrm{D}}^{(m)}\right)^2$ on the $\left(\hat{F}_{\mathrm{ST}}^{(m)}\right)^2$ and measure $\mathrm{R^2}_{\mathrm{QUADRATIC}}$. Both are reported in Tables D.6 and D.7 for all the analyses with real and simulated phenotypes.

To test whether there is a significant difference between $\mathrm{R^2}_{\mathrm{LINEAR}}$ and $\mathrm{R^2}_{\mathrm{QUADRATIC}}$ we perform a permutation two-sample t-test as described in [3], using 10000 permutations. The resulting p-value is 0.784, hence we conclude that the difference between the relationship we consider in this paper and that suggested in [4] is not significant.

Data	Trait	Training Population	R^2_{LINEAR}	$R^2_{\text{QUADRATIC}}$
WHEAT	Yield	France	0.575	0.634
	Height	France	0.371	0.424
	Flowering Time	France	0.412	0.410
	Grain protein content	France	0.681	0.681
MICE	Weight	F005	0.056	0.064
		F008	0.246	0.236
		F010	0.537	0.463
		F016	0.311	0.242
	Growth	F005	0.446	0.437
		F008	0.426	0.404
		F010	0.013	0.019
		F016	0.384	0.372

Table D.6. R^2_{LINEAR} and $R^2_{\text{QUADRATIC}}$ for the data analyses on real phenotypes.

Simulation	Sample Size	Causal Variants	R^2_{LINEAR}	$R^2_{\text{QUADRATIC}}$
Genomic selection	200	10	0.387	0.358
	200	50	0.307	0.307
	200	200	0.122	0.112
	200	1000	0.263	0.261
	800	800	0.284	0.293
	800	1000	0.351	0.352
Cross-population	435	5	0.123	0.093
	435	20	0.175	0.167
	435	100	0.565	0.496
	435	2000	0.131	0.116
	435	10000	0.023	0.035
	435	50000	0.256	0.118

Table D.7. R^2_{LINEAR} and $R^2_{\text{QUADRATIC}}$ for the data used in the simulation studies.

References

[1] Benjamini, Y. and Hochberg, Y. (1995). Controlling the False Discovery Rate: A Practical and Powerful Approach to Multiple Testing. *J. Roy. Stat. Soc. B*, 57(1):289–300.

[2] Hotelling, H. (1953). New Light on the Correlation Coefficient and Its Transforms. *J. Roy. Stat. Soc. B*, 15(2):193–232.

[3] Pesarin, F. and Salmaso, L. (2010). *Permutation Tests for Complex Data: Theory, Applications and Software*. Wiley.

[4] Pszczola, M., Strabel, T., Mulder, A., and Calus, M. P. L. (2012). Reliability of Direct Genomic Values for Animals with Different Relationships within and to the Reference Population. *J. Dairy Sci.*, 95(1):389–400.
